# Age-related changes in estradiol and longitudinal associations with fat mass in men

**DOI:** 10.1371/journal.pone.0201912

**Published:** 2018-08-02

**Authors:** Albert Wu, Zumin Shi, Sean Martin, Andrew Vincent, Leonie Heilbronn, Gary Wittert

**Affiliations:** Freemasons Foundation Centre for Men’s Health, Discipline of Medicine, School of Medicine, University of Adelaide, Adelaide, Australia; University of Colorado Denver School of Medicine, UNITED STATES

## Abstract

**Context:**

In men, circulating 17β-estradiol originates primarily from peripheral aromatization of testosterone particularly in adipose tissue. The effect of ageing and obesity on circulating estradiol remains unclear.

**Objective:**

Determine five-year changes in serum estradiol and the association with testosterone and fat mass in Australian men.

**Design:**

Longitudinal cohort study. At baseline and five-year follow-up, socio-demographic and health-related data including behaviors, chronic conditions, and medication use were collected by questionnaire. Estradiol and testosterone were assayed by liquid chromatography-tandem mass spectrometry and sex hormone-binding globulin by immunochemiluminescent assay. Fat mass was assessed by dual-energy X-ray absorptiometry.

**Participants:**

Community-dwelling men aged 35 years and older at enrollment, resident in the northern and western suburbs of Adelaide without established disease of, or medications affecting, the hypothalamus-pituitary-gonadal axis (n = 725).

**Main outcome measures:**

The dependence of change in serum estradiol over five years on age, testosterone and fat mass after adjustment for multiple confounders.

**Results:**

At baseline, mean age was 53.0 ± 10.8 years. Mean serum estradiol levels at baseline and five-year follow-up were 94.9 ± 34.8 and 89.4 ± 30.4 pmol/L respectively (-1.1 pmol/L/year). On multivariable analyses, estradiol change was associated with changes in testosterone (B-estimate = 2.719, standard error = 0.369, p˂0.001), but not age or total fat mass. Change in testosterone/estradiol ratio was inversely associated with change in fat mass (B = -1.450, SE = 0.575, p = 0.012), and this was consistent across quartiles of fat mass change.

**Conclusions:**

In healthy men, circulating estradiol is primarily dependent on testosterone. With increasing fat mass, estradiol decreases less than testosterone. From a clinical standpoint these data indicate that obesity is associated with a change in the testosterone to estradiol ratio, but a change in estradiol does not occur unless some other pathology is present.

## Introduction

Most circulating 17β-estradiol in men is produced from aromatization of testosterone, predominantly in adipose tissue [1]. At present, the relative effects of age, fat mass and testosterone on estradiol are unclear.

Some previous studies measured estradiol using immunoassays which are inaccurate at the low levels found in men [2]. In cross-sectional studies which used immunoassays, estradiol levels have been variably reported to increase [3], decrease [4, 5] or remain unchanged [6, 7] with increasing age. Similarly in cross-sectional studies which used mass spectrometry, estradiol levels have been reported to increase [8, 9], remain stable [10] or decrease [11–13] with increasing age. A number of longitudinal studies have published data on longitudinal estradiol changes; however, they either do not specifically report the association between estradiol and age, or focus on bone health or mortality rather than adipose tissue [14, 15]. As far as we can determine, the only study which investigated the longitudinal change in estradiol with aging and incorporated body mass index (BMI) found that there was no change with aging [16]. This study used immunoassays to measure estradiol.

Some previous studies have been limited by the use of BMI which is an inaccurate measure of fat mass especially in young men because BMI is also affected by muscle mass. Cross-sectional studies which used immunoassays to measure estradiol found positive [3, 5, 7, 17, 18] and no association [6] between estradiol and BMI, and positive [17–20] and no association [4, 6, 21] between estradiol and fat mass. Cross-sectional studies which used mass spectrometry found positive [8, 9, 13, 22, 23] and no association [12] between estradiol and BMI, and positive association [22, 23] between estradiol and fat mass. One longitudinal study which used immunoassays found no association between estradiol and BMI [16].

Thus, there are limitations in the currently published data on the associations between estradiol, and age and fat mass. To our knowledge, there are no studies which simultaneously use 1) longitudinal estradiol data measured using mass spectrometry, and 2) a direct measure of fat mass. We therefore determined, in a cohort of middle-aged and elderly men, the annualized changes in estradiol with age, fat mass and testosterone over a five-year period. Our hypothesis was that the change in serum estradiol over time follows any change in serum testosterone, an effect modified by fat mass. To test these hypotheses, we performed a 28-day overfeeding study on men and measured the change in adipose tissue aromatase expression.

## Methods

### The Florey Adelaide Male Ageing Study (FAMAS) cohort

The details of FAMAS have been previously published [24]. Briefly, FAMAS is a longitudinal cohort study of men residing in the community in the North West Suburbs of Adelaide and aged 35–80 years at enrollment. Data were collected from 1195 men at baseline (2002–2005) and from 950 men at five-year follow-up (2007–2010). Institutional review board approval was obtained and informed consent obtained from all participants.

**Fig 1:**
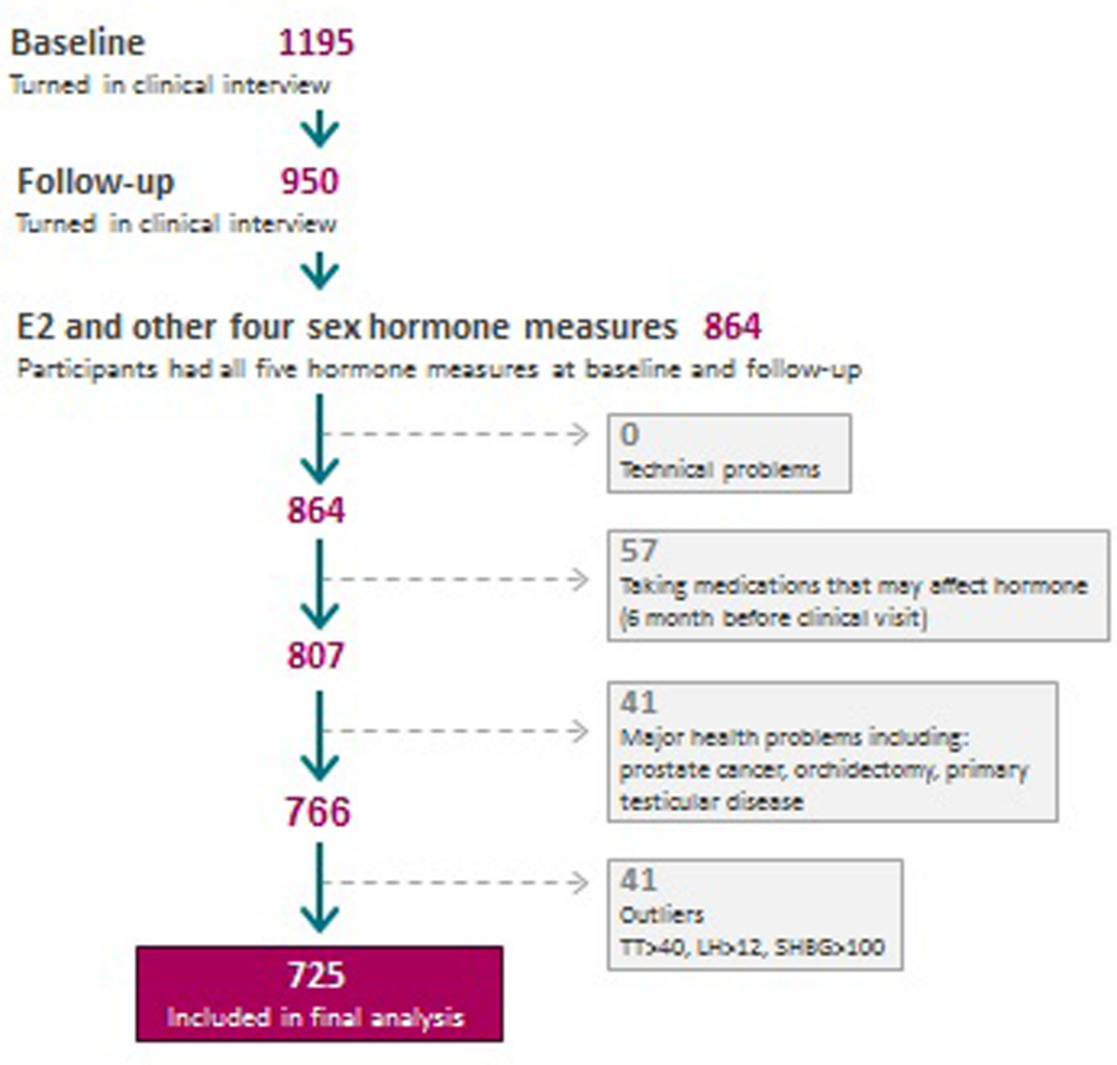
Selection of sample for analysis.

The analytic sample was selected as shown in Fig 1. Men who were taking a medication known to affect the hypothalamo-pituitary-gonadal (HPG) axis (testosterone, antiandrogen, glucocorticoid, opioid, antiepileptic, antipsychotic, 5-α reductase inhibitor, aromatase inhibitor) currently or within the 6 months prior to the clinic visit were excluded. The final sample for analysis included 725 men (Fig 1).

Data on age, marital status, employment, health-related behaviors (e.g. smoking, alcohol, and physical activity), current and past health problems, and medication use were obtained by questionnaire [24]. Symptoms of depression were assessed using Beck Depression Inventory-IA [25]. Depression was defined as score ≥ 13. The presence of type 2 diabetes mellitus (T2DM) was determined by self-reported clinician diagnosis, fasting plasma glucose ≥ 7.0 mmol/L or HbA1c ≥ 6.5%. Medication use was confirmed by data linkage with a national medication registry. During each clinic visit, weight, height and waist circumference were measured, and BMI calculated as previously described [24]. Whole and regional body composition was measured by dual-energy x-ray absorptiometry (DXA) performed either on a fanbeam (Prodigy DF + 14759, Encore software version 9.15) or pencil-beam (DPX+, Lunar software version 4.7e) densitometer (both machines from GE Lunar, Madison, WI, USA), with identical protocols followed for both densitometers and both time points. Total fat mass, and lean mass (excludes bone mass) in kilograms were defined for whole body using default settings. For assessment of soft tissue composition in the abdominal region, the top of lumbar vertebrae L2 to the bottom of L4 and extending outward to a vertical line touching the inner edges of the rib cage was adopted as the customized anatomical setting. Percentage abdominal fat mass was computed as [abdominal fat mass/(abdominal fat mass + abdominal lean mass)] * 100. Previously published data demonstrated no significant differences between densitometers [26]. Additional validation studies were conducted in our laboratory, with means for body composition measures (fat mass, lean mass, bone mineral content, and bone mineral density) obtained using both densitometers in a subsample of FAMAS participants (n = 18; age range, 44–70 years; body mass range, 56–121 kg) found to be highly correlated (all r > 0.95); and small systematic differences were detected (weaker correlations, but all r > 0.82) for lean mass, bone mineral content, and fat mass percentage for older (<55 years vs 55+ years) and heavier men (<87 kg vs 87+ kg).

Morning fasting blood samples were obtained by venipuncture and stored at -80°C and assays were carried out as previously described [27]. Briefly, validated stable isotope dilution LC-MS was used to measure total testosterone (interassay coefficient of variation/CV 9.3% at 0.43 nmol/L, 8.6% at 1.66 nmol/L, 4.0% at 8.17 nmol/L) and estradiol (interassay CV 14% at 23 pmol/L, 4.0% at 83 pmol/L, 6.0% at 408 pmol/L). Immunoassays were used to measure SHBG (interassay CV 4% at 32.3 nmol/L), follicle-stimulating hormone (FSH, interassay CV 3.1% at 7.0 U/L) and luteinizing hormone (LH, interassay CV 4.0% at 7.7 U/L). Plasma glucose was measured using an automated chemistry analyzer system (interassay CV 2.5% at 3.5 mmol/L, 3.0% at 19.6 mmol/L). Glycated hemoglobin (HbA1c) was measured by high-pressure liquid chromatography using a spherical cation exchange gel (CV 2% at 6% of total hemoglobin).

Annualized changes in measured parameters were determined by dividing the difference between baseline and follow-up values by the number of years between visits. Multivariable regressions were built for annualized change in estradiol and testosterone/estradiol ratio according to baseline and change in health factors. Independent variables that were considered to influence estradiol levels were selected based on previous studies and the authors’ judgement. Additional analyses were performed with SHBG removed from regressions due to multicollinearity between SHBG and fat mass. The statistics software R was used and p < 0.05 (two-tailed) was considered significant.

### Adipose tissue aromatase expression in response to overfeeding

Adipose tissue aromatase mRNA was measured using cDNA remaining from 8 men without diabetes or cardiovascular disease who were participants in a 28-day overfeeding study. This study has previously been described [28]. Exclusion criteria included weight change of >2 kg over the preceding 6 months, >60 minutes of exercise per week, medications affecting insulin sensitivity or blood pressure. Institutional Human Ethics Review Board approval was obtained. All participants provided signed informed consent. This study is registered as a clinical trial at clinicaltrials.gov (registration number NCT00562393).

From day -3 to day 0, participants were provided with their baseline energy requirements for weight maintenance (30% fat, 15% protein and 55% carbohydrate). On days 0–3 and 25–28, participants were provided with baseline energy requirements plus 5,200 kJ/day (45% fat, 15% protein and 40% carbohydrate). On days 3–25, participants were instructed to consume their regular diets with supplemental food provided to achieve an intake of 5,200 kJ/day above baseline energy requirements. Participants were weighed and compliance monitored weekly by the research nurse and dietitian.

At baseline and after 28 days overfeeding, fat mass was measured by DXA as above (Lunar DPX GE Lunar, Lunar Corp, Madison, WI). Serum testosterone and estradiol were measured by a validated stable-isotope dilution LC-MS/MS [29]. A sample of periumbilical subcutaneous adipose tissue was obtained using needle biopsy [30]. Total RNA was extracted from 100–150 mg of adipose tissue using TRIzol reagent, and cDNA was synthesized using Omniscript RT kit and Recombinant RNAsin RNase inhibitor (Qiagen). Inventoried Taqman assays for Cyp19a and 18S were purchased from Life Technologies. qPCR was performed in duplicate, with negative controls, using the manufacturer’s recommended conditions on a 7500 Fast Real-Time PCR system (Applied Biosystems). Data were analyzed using the 2^−ΔCt^ method and an internal single reference gene, 18S. Statistical analysis was performed using R. Data at different time points were compared using paired t-tests. p < 0.05 was considered significant.

## Results

The characteristics of the sample at baseline and follow-up are shown in Tables 1 and Table 2 shows changes in health factors during follow-up. At baseline, the mean age was 53.0 ± 10.8 years, 30.2% of the sample were obese (BMI ≥ 30), 20.2% were current smokers, 8.1% had T2DM and 6.8% had CVD. Mean serum estradiol levels at baseline and follow-up were 94.9 ± 34.8 and 89.4 ± 30.4 pmol/L respectively, a change of -1.1 pmol/L/y.

**Table 1:** Sample characteristics by study waves (n = 725).

Health factors	Baseline^a^	Follow-up
Age (years)	53.0 ± 10.8	58.0 ± 10.8
Sex hormones		
estradiol (pmol/L)	94.9 ± 34.8	89.4 ± 30.4
total testosterone (nmol/L)	17.2 ± 5.5	16.2 ± 5.2
sex hormone-binding globulin (nmol/L)	33.1 ± 13.3	37.0 ± 13.9
luteinizing hormone (U/L)	4.9 ± 2.3	4.3 ± 2.0
follicle-stimulating hormone (U/L)	7.0 ± 5.6	7.7 ± 5.6
Percentage total fat mass	27.1 ± 6.9	28.8 ± 6.4
n/a^b^ (n)	49	46
Percentage abdominal fat mass	33.8 ± 7.9	36.3 ± 8.1
n/a (n)	49	49
Marital status		
married/partnered	598 (82.7%)	573 (81.9%)
no	125 (17.3%)	127 (18.1%)
n/a	2	25
Employment status		
employed	493 (68.1%)	422 (60.1%)
unemployed	13 (1.8%)	17 (2.4%)
retired	158 (21.8%)	216 (30.8%)
other	60 (8.3%)	47 (6.7%)
n/a	1	23
Smoking status		
non-smoker	266 (36.8%)	269 (37.8%)
ex-smoker	310 (42.9%)	325 (45.7%)
current smoker	146 (20.2%)	117 (16.5%)
n/a	3	14
Body mass index (kg/m^2^)	28.4 ± 4.2	28.7 ± 4.4
Body mass index (kg/m^2^)		
< 25	147 (20.3%)	135 (18.6%)
25–30	359 (49.5%)	354 (48.8%)
≥ 30	219 (30.2%)	236 (32.6%)
Waist circumference (cm)		
< 95	238 (32.8%)	272 (37.6%)
95–100	141 (19.4%)	123 (17.0%)
≥ 100	346 (47.7%)	329 (45.4%)
n/a	0	1
Depression		
no	648 (92.6%)	622 (91.7%)
yes	52 (7.4%)	56 (8.3%)
n/a	25	47
Type 2 diabetes mellitus		
no	666 (91.9%)	610 (84.1%)
yes	59 (8.1%)	115 (15.9%)
Cardiovascular disease		
no	676 (93.2%)	661 (91.2%)
yes	49 (6.8%)	64 (8.8%)

^a^Data expressed as mean ± standard deviation, or n (%)

^b^n/a, not measured or reported

**Table 2:** Changes in health factors between baseline and follow-up (n = 725).

Changes in health factors	n (%)
Married/partnered	
not married/partnered at both times	101 (14.4%)
became married/partnered	20 (2.9%)
no longer married/partnered	25 (3.6%)
married/partnered at both times	553 (79.1%)
n/a^a^	26
Retired during follow-up	
no	634 (90.3%)
yes	68 (9.7%)
n/a	23
Smoking status	
non-smoker at both times	552 (77.7%)
became a smoker	14 (2.0%)
no longer a smoker	41 (5.8%)
smoker at both times	103 (14.5%)
n/a	15
Depression	
not depressed at both times	584 (88.6%)
became depressed	28 (4.2%)
no longer depressed	21 (3.2%)
depressed at both times	26 (3.9%)
n/a	66
Type 2 diabetes mellitus	
no diabetes at both times	610 (84.1%)
developed diabetes	56 (7.7%)
had diabetes at baseline	59 (8.1%)
Cardiovascular disease	
no disease at both times	646 (89.1%)
developed disease	30 (4.1%)
had disease at baseline	49 (6.8%)
Annualized change in sex hormones^b^	
estradiol (pmol/L)	-1.1 (-1.6, -0.6)
total testosterone (nmol/L)	-0.2 (-0.3, -0.2)
sex hormone-binding globulin (nmol/L)	0.8 (0.6, 0.9)
luteinizing hormone (U/L)	-0.1 (-0.2, -0.1)
follicle-stimulating hormone (U/L)	0.1 (0.1, 0.2)
Annualized change in percentage total fat mass^b^	0.3 (0.3, 0.4)
n/a	88
Annualized change in percentage abdominal fat mass^b^	0.5 (0.4, 0.6)
n/a	90

^a^n/a, not measured or reported

^b^Data expressed as mean (95% confidence interval)

In multivariable regression of annualized change in estradiol, estradiol change was not predicted by baseline age, total percentage fat mass or testosterone (Table 3). There was, however, a strong association of annualized change in estradiol with testosterone change but not with fat mass change or age (Table 4). Change in SHBG was inversely associated with the change in estradiol (p = 0.005), but this was no longer significant after testosterone was removed from the regression (estimate = 0.061, p = 0.685). The association of change in estradiol with testosterone change remained significant after SHBG was removed (estimate = 2.264, p < 0.001).

**Table 3:** Multivariable regression for annualized change in estradiol according to baseline health factors. n = 651 due to missing data. R^2^ = 0.020; adjusted R^2^ = 0.005.

	Annualized change in estradiol (pmol/L)	
Baseline health factors	Estimate	p-value
Age (years)	-0.035	0.237
Total testosterone (nmol/L)	-0.086	0.187
Sex hormone-binding globulin (nmol/L)	0.046	0.107
Marital status		
married/partnered	0.000	
no	-0.298	0.676
Smoking status		
non-smoker	0.000	
ex-smoker	0.292	0.618
current smoker	-0.408	0.575
Percentage total fat mass	-0.011	0.788
Depression		
no	0.000	
yes	1.648	0.104
Type 2 diabetes mellitus		
no	0.000	
yes	-1.063	0.285
Cardiovascular disease		
no	0.000	
yes	-1.962	0.070

*p < 0.05

**Table 4:** Multivariable regression for annualized change in estradiol according to changes in health factors. n = 576 due to missing data. R^2^ = 0.116; adjusted R^2^ = 0.087.

	Annualized change in estradiol (pmol/L)	
Changes in health factors	Estimate	p-value
Age (years)	-0.006	0.821
Annualized change in testosterone (nmol/L)	2.719	< 0.001*
Annualized change in SHBG (nmol/L)	-0.453	0.005*
Married/partnered		
not married/partnered at both times	0.000	
became married/partnered	-3.144	0.082
no longer married/partnered	-1.244	0.421
married/partnered at both times	-0.410	0.593
Retired during follow-up		
no	0.000	
yes	0.011	0.990
Smoking status		
non-smoker at both times	0.000	
became a smoker	0.060	0.975
no longer a smoker	-0.094	0.931
smoker at both times	-0.329	0.676
Annualized change in percentage total fat mass	0.242	0.393
Depression		
not depressed at both times	0.000	
became depressed	-0.903	0.468
no longer depressed	0.149	0.919
depressed at both times	1.777	0.190
Type 2 diabetes mellitus		
no diabetes at both times	0.000	
developed diabetes	-0.246	0.800
had diabetes at baseline	-1.885	0.064
Cardiovascular disease		
no disease at both times	0.000	
developed disease	1.693	0.217
had disease at baseline	-1.837	0.098

*p < 0.05

**Fig 2:**
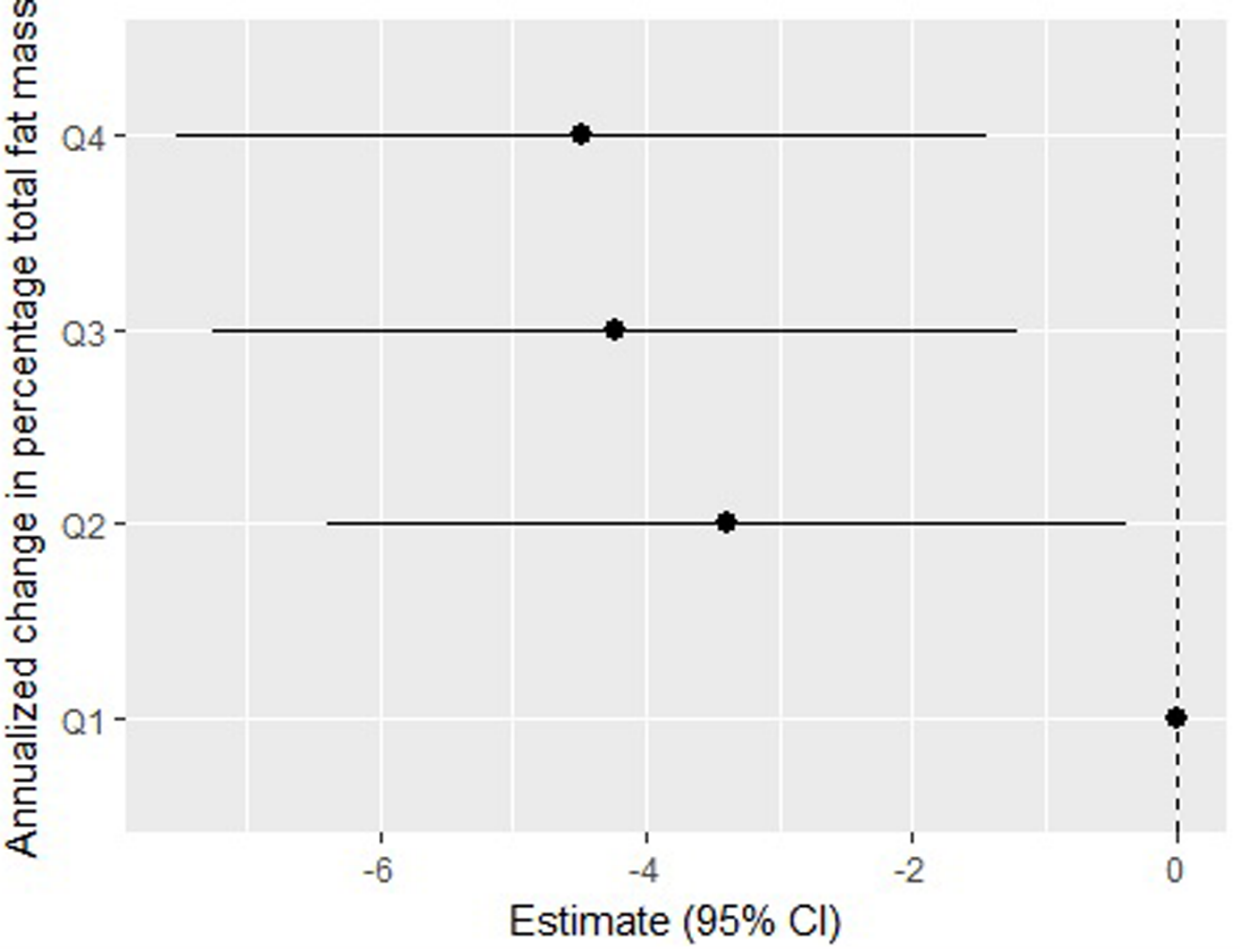
Trends in annualized change in testosterone/estradiol ratio according to quartiles of fat mass change after adjustment for changes in health factors apart from SHBG. n = 576 due to missing data. R^2^ = 0.058; adjusted R^2^ = 0.027.

In multivariable regression, the annualized change in testosterone/estradiol ratio was not associated with annualized change in percentage total fat mass (estimate = -0.763, p = 0.179). The association became significant after SHBG was removed from the regression (estimate = -1.450, p = 0.012) (Table 5). In addition, this inverse relationship between testosterone/estradiol change and change in fat mass was consistent across quartiles of percentage total fat mass change (Fig 2).

**Table 5:** Multivariable regression for annualized change in testosterone/estradiol ratio according to changes in health factors. n = 576 due to missing data. R^2^ = 0.051; adjusted R^2^ = 0.023.

	Annualized change in testosterone/estradiol	
Changes in health factors	Estimate	p-value
Age (years)	-0.018	0.753
Married/partnered		
not married/partnered at both times	0.000	
became married/partnered	3.323	0.374
no longer married/partnered	1.771	0.580
married/partnered at both times	3.824	0.016*
Retired during follow-up		
no	0.000	
yes	-0.203	0.915
Smoking status		
non-smoker at both times	0.000	
became a smoker	3.389	0.395
no longer a smoker	-3.142	0.161
smoker at both times	1.966	0.227
Annualized change in percentage total fat mass	-1.450	0.012*
Depression		
not depressed at both times	0.000	
became depressed	2.991	0.246
no longer depressed	4.272	0.160
depressed at both times	-2.410	0.391
Type 2 diabetes mellitus		
no diabetes at both times	0.000	
developed diabetes	2.209	0.272
had diabetes at baseline	2.236	0.288
Cardiovascular disease		
no disease at both times	0.000	
developed disease	-3.056	0.282
had disease at baseline	2.067	0.369

*p < 0.05

When all the above analyses were repeated using percentage abdominal fat mass instead of percentage total fat mass, the associations remained similar (data not shown). The inverse relationship between changes in testosterone/estradiol and fat mass remained strong when percentage abdominal fat mass was used as a continuous variable (estimate = -1.269, p = 0.007) but the relationship was not consistent across quartiles of fat mass change.

### Overfeeding study

**Fig 3:**
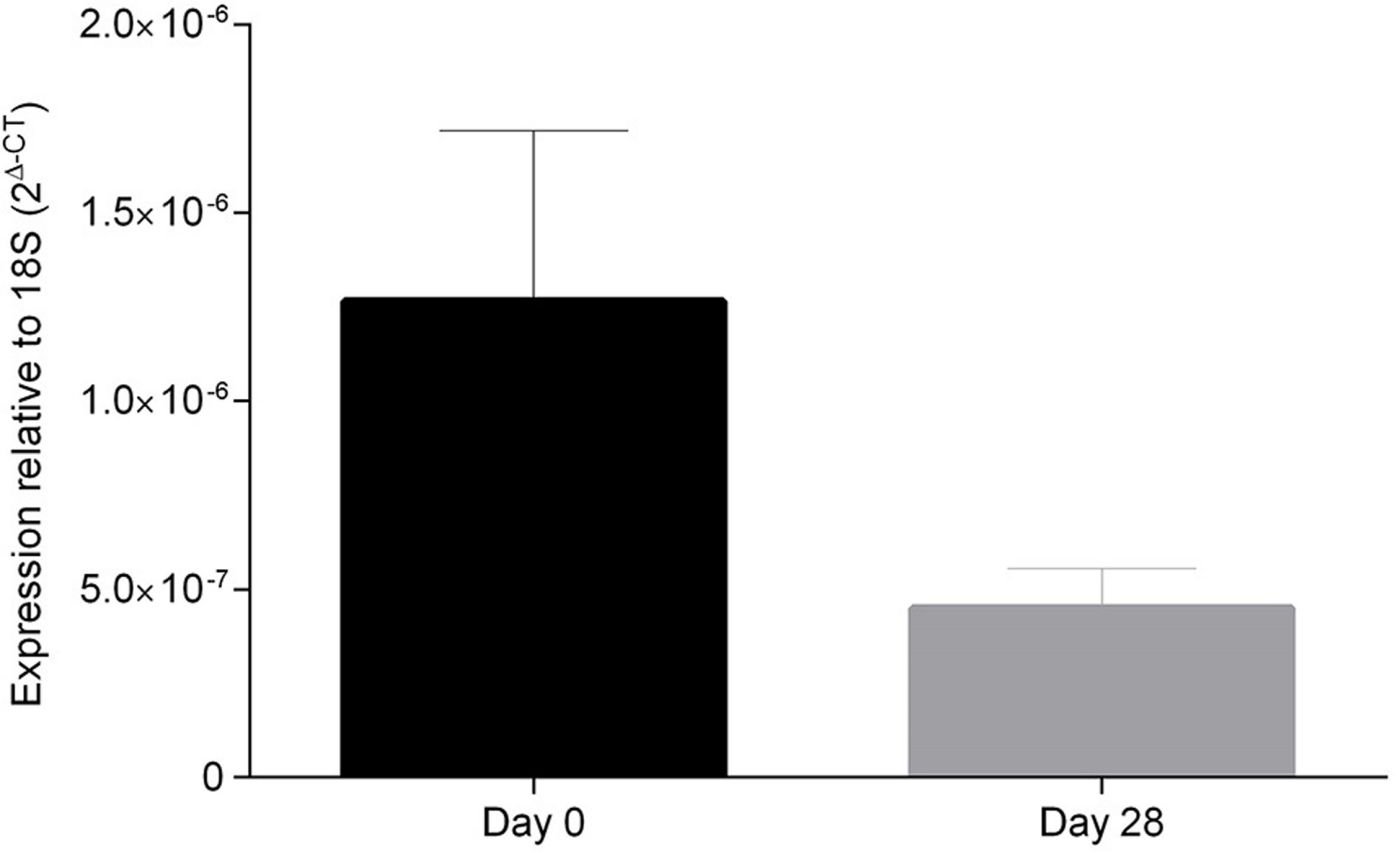
Aromatase mRNA expression in subcutaneous adipose tissue during 28 days overfeeding experiment.

The 8 men had a mean age of 36.1 ± 6.6 years and BMI of 26.8 ± 3.5 kg/m^2^. During the 28 days of overfeeding, body weight increased from 83.9 ± 12.2 kg to 86.2 ± 12.2 kg (p = 0.009) and percentage fat mass increased from 29.6 ± 7.7 to 31.1 ± 7.4 (p = 0.032). Aromatase mRNA expression did not change (p = 0.086) (Fig 3). Serum estradiol, testosterone and testosterone/estradiol ratio did not change (p = 0.907, p = 0.268, p = 0.773 respectively).

## Discussion

Using longitudinal data from a large sample of men, we found that estradiol change was not independently associated with increasing age or fat mass. However, estradiol change did have a strong direct association with testosterone change, suggesting that estradiol levels are predominantly dependent on testosterone. In addition, testosterone to estradiol conversion had a direct association with change in fat mass. In the overfeeding study, we found no change in adipose tissue aromatase expression with weight gain.

Our finding that estradiol remains stable with increasing age is consistent with previous longitudinal data from the Framingham Heart Study which used immunoassay to measure estradiol [16]. Our data are also consistent with previous cross-sectional studies which used immunoassay [6, 7] and pooled data (n = 10,904) using mass spectrometry from three large observational studies in Australia [10]. In contrast, some cross-sectional studies have found that estradiol increases with aging using immunoassay [3] and mass spectrometry [8, 9], and others have found that estradiol decreases with aging using immunoassay [4, 5] and mass spectrometry [11–13]. The reasons for these discrepancies are unclear and cannot be entirely attributed to the use of immunoassay.

Among studies that contained longitudinal estradiol data [14, 15], one investigated the association between BMI and estradiol [16], and found no association. Using more precise measurements of fat mass and estradiol, we similarly find no association between changes in estradiol and fat mass. In contrast, we observe a strong independent relationship between changes in estradiol and testosterone suggesting that a falling testosterone level with increasing age is the primary determinant of change in estradiol. Our data also show that the rate of decline of estradiol is less than the rate of decline in testosterone, and this can partly be explained by the degree of obesity.

Studies have found an association between fat mass and testosterone to estradiol conversion. For example, studies have demonstrated that weight loss in men leads to decreases in estradiol and testosterone to estradiol conversion [31, 32]. In addition, in men administered testosterone, older men had higher estradiol and estradiol/testosterone ratio than younger men, which could be partly explained by the higher percentage fat mass in older men [33]. One possible explanation for these observations is that with the increased mass of adipose tissue, the overall aromatase activity is increased. An alternative explanation is that there is an increased expression of aromatase per unit of adipose tissue. To investigate these possibilities, we examined the effect of overfeeding 8 men an energy dense diet for 28 days, and found no change in aromatase expression in response to acute experimental overfeeding and weight gain. Therefore, we propose that increase in total fat mass is accompanied by an increase in overall aromatase activity which reduces the fall in estradiol relative to testosterone. We cannot exclude the possibility that aromatase expression increases in the visceral adipose tissue; however, we did find that testosterone to estradiol conversion was independently associated with both percentage total as well as abdominal fat mass.

The study cohort consisted of urban Adelaide-dwelling primarily Caucasian men which may limit generalizability of the findings to other ethnicities and geographic regions. A further limitation of this study is the observational nature which precludes any direct inferences about causality. Furthermore, the overfeeding experiment had limited subjects which limits the power of its findings.

In summary, using a longitudinal study of men, we found that estradiol change was predominantly dependent on change in testosterone rather than ageing or fat mass. Testosterone to estradiol conversion increased with increasing fat mass, possibly due to increased overall aromatase activity.

## Supporting information

S1 Table Main study data set. (XLSX)

S2 Table Overfeeding study data set. (XLSX)
